# Supplementary material for: Carrot DcALFIN4 and DcALFIN7 Transcription Factors Boost Carotenoid Levels and Participate Differentially in Salt Stress Tolerance When Expressed in Arabidopsis thaliana and Actinidia deliciosa
Source: Int J Mol Sci. 2022 Oct 12;23(20):12157. doi: 10.3390/ijms232012157 (PMC9603649; doi:10.3390/ijms232012157)
Supplement: Supplementary file 1 [file ijms-23-12157-s001.zip › ijms-1924677-supplementary.pdf]

Carrot DcALFIN4 and DcALFIN7 Transcription Factors Boost Carotenoid Levels and Participate Differentially in Salt Stress Tolerance When Expressed in *Arabidopsis thaliana* and *Actinidia deliciosa*

Luis Felipe Quiroz-Iturra [1], Kevin Simpson [2], Daniela Arias [3], Cristóbal Silva [3], Christian González-Calquin [3],
Leticia Amaza [3], Michael Handford [3] and Claudia Stange [3,*]

[1] Genetics & Biotechnology Lab, Plant & AgriBiosciences Research Centre (PABC), Ryan Institute, University of Galway, University Road, H91 REW4 Galway, Ireland; luis.quiroz@universityofgalway.ie

[2] Departamento de Genética Molecular y Microbiología, Facultad de Ciencias Biológicas, Pontificia Universidad Católica de Chile, Alameda 340, Santiago 7750000, Chile; kevinsimpson@ug.uchile.cl

[3] Departamento de Biología, Facultad de Ciencias, Universidad de Chile, Las Palmeras 3425, Ñuñoa, Santiago 7750000, Chile; danielaloreto.arias@gmail.com (D.A.); cristobal.silvam@usach.cl (C.S.); christian.gonzalez@usach.cl (C.G.-C.); letty.amaza@gmail.com (L.A.); mhandfor@uchile.cl (M.H.)

* Correspondence: cstange@uchile.cl; Tel.: +56-22-2978-7361

Table S1: List of primers used in this study

Name	Sequence	Tm (°C)	Size (pb)	Use
Oligo AP	CGCCACGCGTCGACTAGTACTTTTTTTTTTTTTTTTT	-	-	cDNA synthesis
DcAlfin4.F	GTGGGGGTTTGTGAAAGAAGGC	58.9	795	Amplify the CDS region of the DcALFIN4 gene without stop codon
DcAlfin4.R	TGGCCTAAGTTTCTTGCTACTGCT	58.5		Amplify the CDS region of the DcALFIN4 gene without stop codon
DcAlfin7.F	TGGCCTAAGTTTCTTGCTACTGCT	59.0	750	Amplify the CDS region of the DcALFIN7 gene without stop codon
DcAlfin7.R	CATATGGACTCTGGCCCTCTTTGA	58.4		Amplify the CDS region of the DcALFIN7 gene without stop codon
qDcAlfin4.F	AGAAGAAGAGGAGCACGGAGACAC	60.1	101	DcALFIN4 qRT-PCR
qDcAlfin4.R	GGAACCACTTCTCACACACATCACAAC	59.7		DcALFIN4 qRT-PCR
qDcAlfin7.F	AATACCCGAGCCAGCATTAG	54.7	90	DcALFIN7 qRT-PCR
qDcAlfin7.R	GAATCACTGTGAACTGCAACAA	53.9		DcALFIN7 qRT-PCR
qDcAREB3.F	GCCAGTTTAGCACCTCGTCTTGC	60.9	179	DcAREB3 qRT-PCR
qDcAREB3.R	CGCCTTCTCTCCACCACTTTCTC	59.5		DcAREB3 qRT-PCR
qDcUbi.F	CAAGACAAAGAAGGCATCCCGC	58.8	177	DcUbi40 qRT-PCR (housekeeping)
qDcUbi.R	CTTGGGCTTGGTGTAGGTCTTC	57.7		DcUbi40 qRT-PCR (housekeeping
qAtPsy.F	GACACCCGAAAGGCGAAAGG	58.8	179	AtPSY qRT-PCR
qAtPsy.R	CAGCGAGAGCAGCATCAA	59.1		AtPSY qRT-PCR
qAtUbi10.F	ACCAGCAGCGTCTCATCTT	56.5	137	AtUbi10 qRT-PCR (housekeeping)
qAtUbi10.R	GCATAACAGAGACGAGATTTAGAA	52.5		AtUbi10 qRT-PCR (housekeeping)
qAtDXS.F	GGCTCGCACGTTGTTCAGTTTC	59.2	77	AtDXS qRT-PCR
qAtDXS.R	GGCAGTACCATTGGTCTCCACTTG	59.7		
qAtDXR.F	TAGGTTGTGCGGGACTAAAGCC	59.3	65	AtDXR qRT-PCR
qAtDXR.R	TGCAAGAGCAATGTCCTTTCCTGC	60.1		
qAd18S.F	CTGTGAAACTGCGAATGGCTC	56.5	116	Ad18s qRT-PCR (housekeeping)
qAd18S.R	TTCCAGAAGTCGGGGTTTGT	52.5		

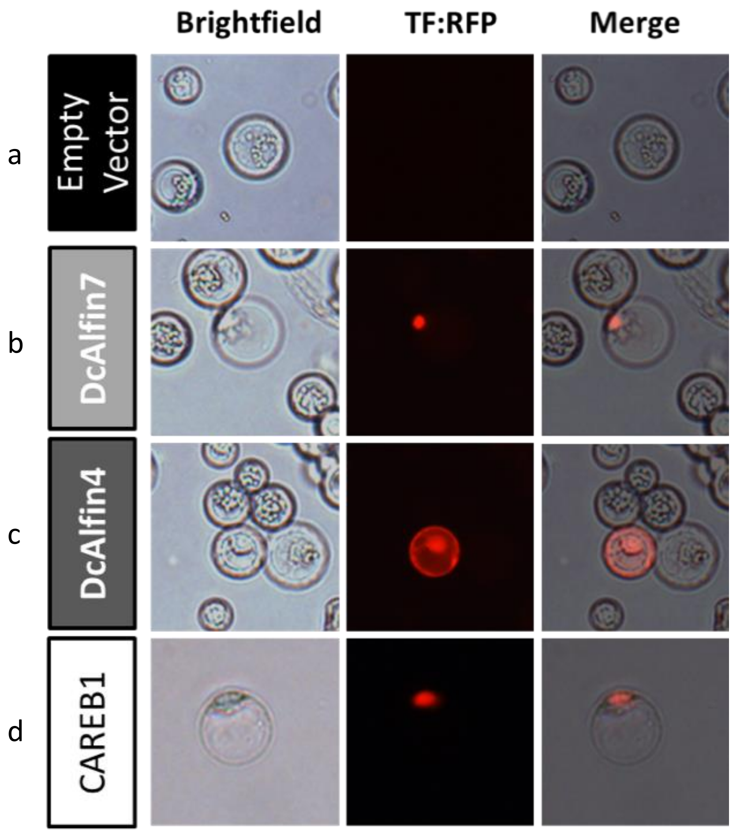

Figure S1. Subcellular localization of DcAL4 and DcAL7 transcription factors in carrot protoplasts. Carrot protoplasts were transfected with pK7RWG2 empty vector and DcALFIN4:RFP and DcALFIN7:RFP vectors. Images were taken 24 hours after transfection. a) pK7RWG2 empty vector, b) DcAL7:RFP, c) DcAL4:RFP and d) CAREB1:RFP. Red channel: Images taken using the Cy3 filter. Bar: 10 µM.

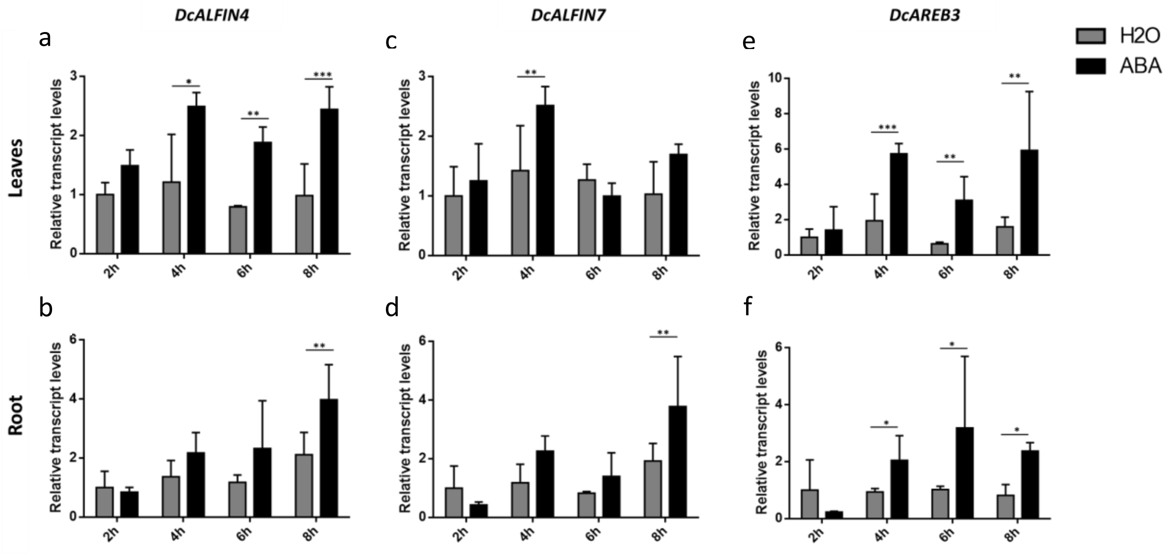

Figure S2. *DcALFIN4* and *DcALFIN7* relative transcript levels in *D. carota* under ABA treatments. *DcALFIN4* relative transcript level in *D. carota* (a) leaves and (b) roots under ABA treatment. *DcALFIN7* relative transcript level in *D. carota* (c) leaves and (d) roots under ABA treatment. *DcAREB3* (treatment positive control) relative transcript level in *D. carota* (e) leaves and (f) roots under ABA treatment. Transcript abundance was normalized to *DcUbiquitin* expression level and the control condition H_2O-2h was taken as calibrator. The relative expression levels were carried out with 3 biological replicates (n=3) and two technical repeats each. Values are means +/-SD. Asterisks indicate statistically significant differences determined by two-tailed ANOVA test: *P < 0.05, **P < 0.01, ***P < 0.001.

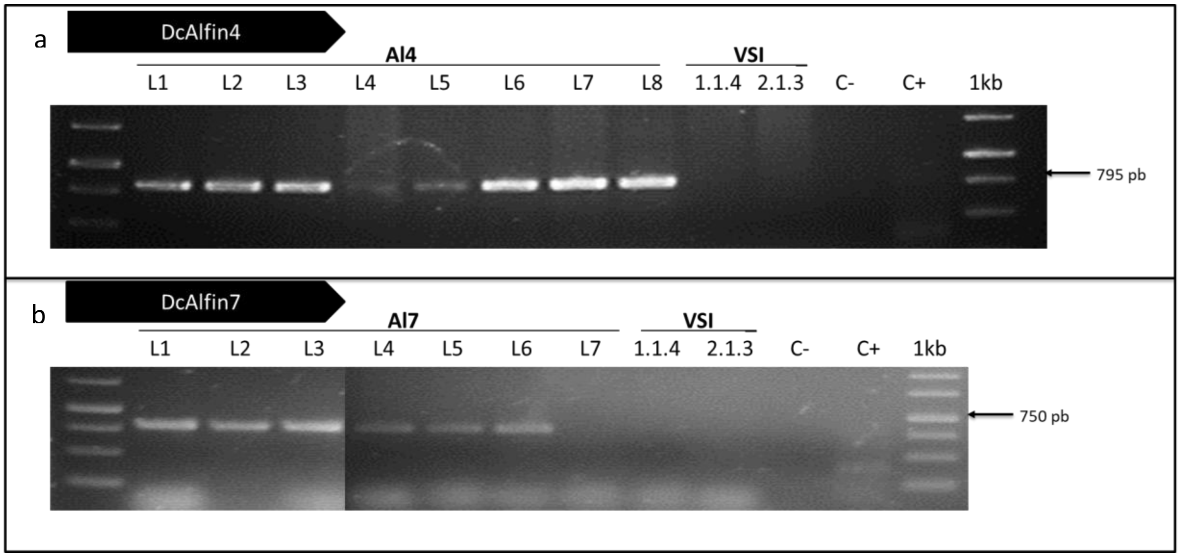

Figure S3. Selection of transgenic T1 *DcALFIN4* and *DcALFIN7 A. thaliana* lines. Amplification of (a) *DcALFIN4* in eight kanamycin-resistant Al4 *A. thaliana* transformants and of (b) seven *DcALFIN7* kanamycin-resistant Al7 *A. thaliana* transformants. C-: PCR negative control (H_2O); C+: PCR positive control (colony of pK7Alfin4- or pK7Alfin7-harboring *A. tumefaciens*); VSI: 1.1.4 and 2.2.3 T3 empty vector lines. The arrow shows the expected size of the respective gene fragment.

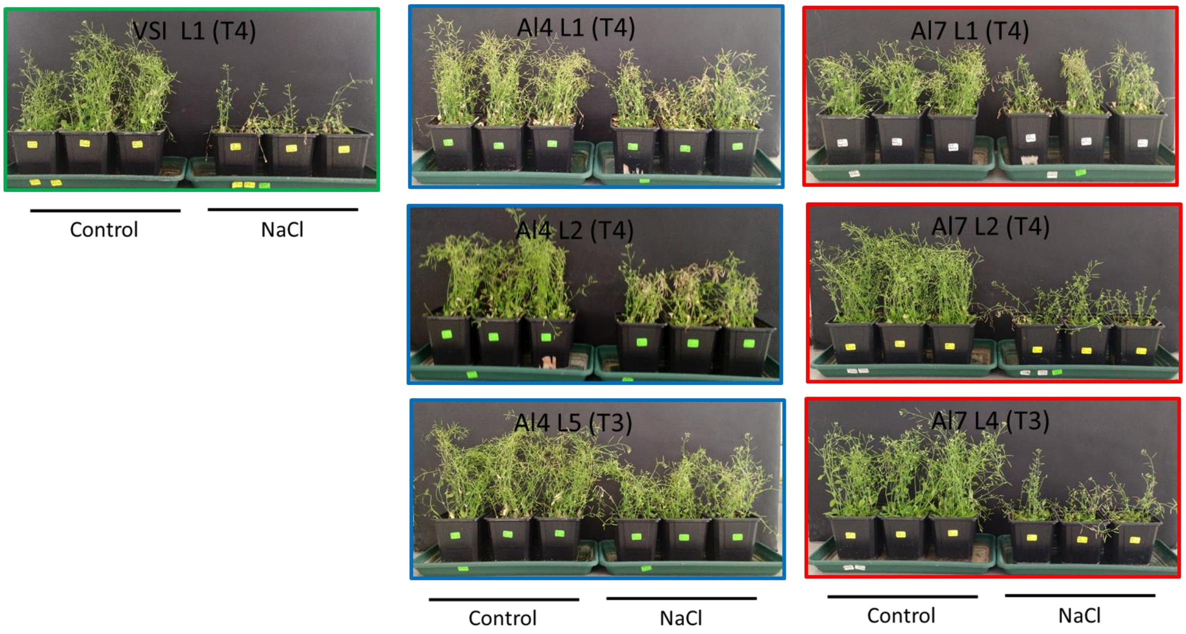

.

Figure S4. Phenotypic response of homozygous EV, AL4 and Al7 *A. thaliana* transgenic lines subjected to chronic salt treatment (200 mM NaCl). Control: Well-watered plants; NaCl: Plants treated with 200 mM NaCl for 14 days followed by 14 days of recovery.

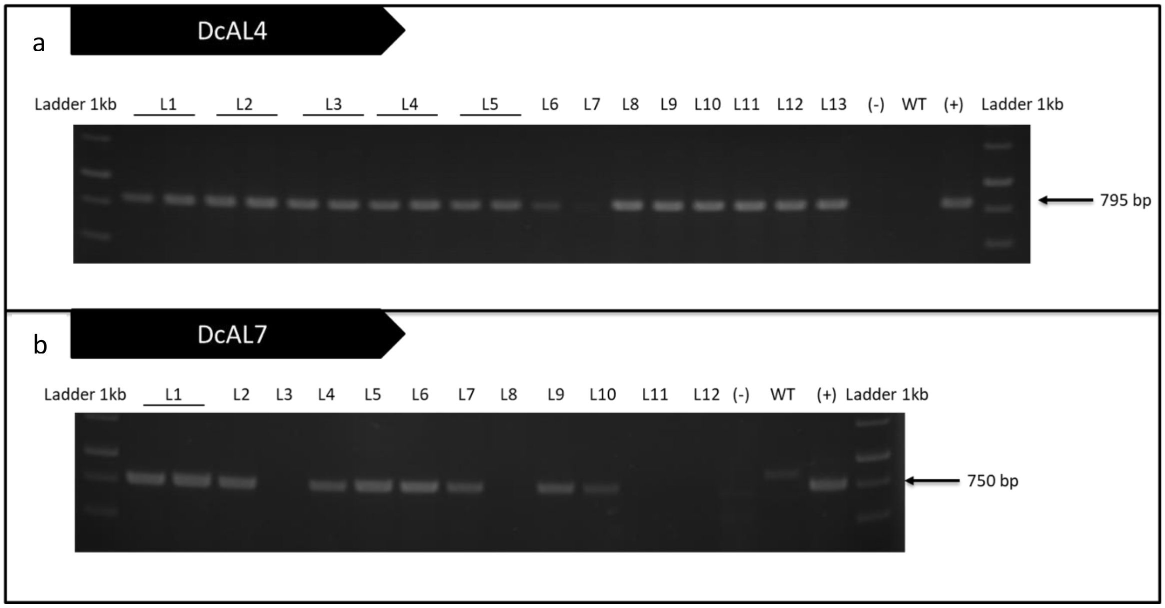

Figure S5. Selection of transgenic T0 *DcALFIN4* and *DcALFIN7 A. deliciosa* lines. Amplification of (a) *DcALFIN4* in thirteen kanamycin-resistant Al4 *A. deliciosa* transformants and of (b) *DcALFIN7* in twelve kanamycin-resistant Al7 *A. deliciosa* transformants. C-: PCR negative control (H_2O); C+: PCR positive control (colony of pK7Alfin4- or pK7Alfin7-harboring *A. tumefaciens*); WT: non transgenic plant. The arrow shows the expected size of the respective gene fragment.

.

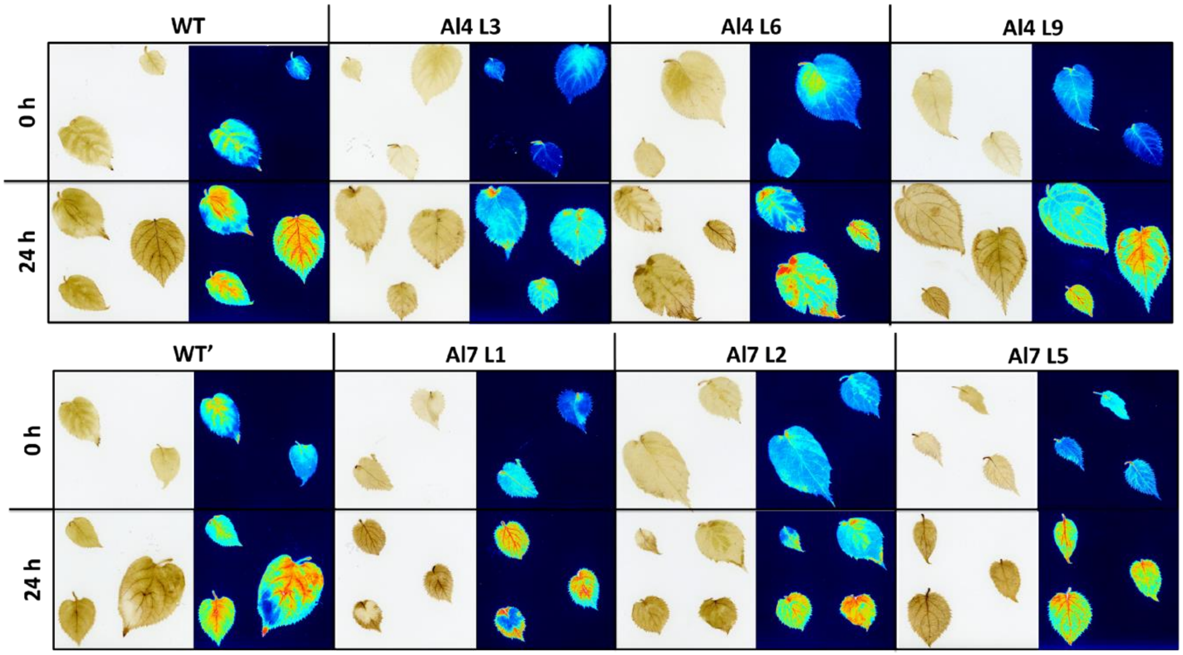

Figure S6. Hydrogen peroxide content in leaves of Al4 and Al7 *A. deliciosa* lines subjected to acute treatment with NaCl. WT, Al4 and Al7 *A. deliciosa* leaves pre and post-treatment with 250 mM NaCl stained with DAB, as well as a false-colored image generated by ImageJ software. The assay was repeated twice.

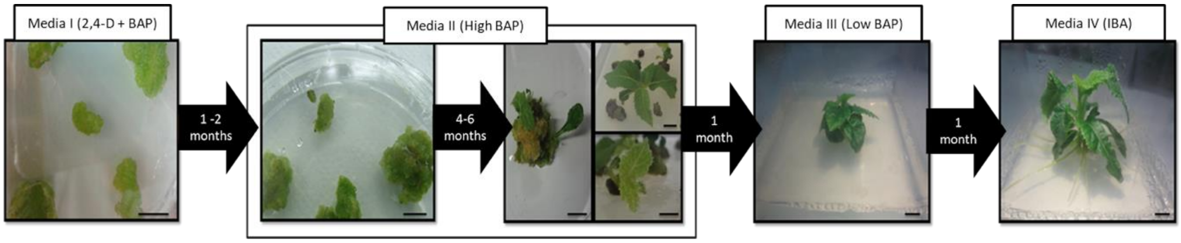

Figure S7. *In vitro* culture and stable transformation of kiwi. Medium I: Callus induction medium supplemented with 2,4-D (2 mg / L) and BAP (0.5 mg / L). Medium II: Callus proliferation and shoot induction medium supplemented with BAP (5 mg / L). Medium III: Shoot proliferation medium without supplementation or with BAP (0.5 mg / L). Medium IV: Rooting medium supplemented with IBA (0.5 mg / L). The arrows indicate the time in each culture medium. Scale bars: 1 cm.

Supplementary Methods I

Protoplast isolation and transfection.

D. carota protoplast isolation was carried out adapting the protocol of [54] and performed according to [13]. Briefly, 50 mL of 4-5-day old *D. carota* cell suspensions were centrifuged at 200 g for 2 minutes. The supernatant was removed and 10 mL of enzyme solution (Cellulase Onozuka R-10 0.75% w / v, 0.25% Macerozyme R-10, 400 mM Mannitol, 10 mM CaCl2, 2.5 mM MES, pH 5.7) was added. The cells were incubated in the dark with agitation (40-50 rpm) for 4-6 h and filtered through a 40 µm nylon membrane. Protoplasts were centrifuged at 60 g for 5 min and the pellet was washed twice in W5 solution (154 mM NaCl, 5 mM KCl, 125 mM CaCl2, 5 mM glucose, pH 5.7) and incubated in W5 on ice for 30 min. Then, the protoplasts were centrifuged at 60 g for 5 min and resuspended in MC solution (5 mM MES, 20 mM CaCl2, 0.5 mM mannitol, pH 5.7). Purified protoplasts were counted using a Neubauer chamber and resuspended at 2x106 protoplasts/mL. The transfection of D. carota protoplasts was performed according to the protocol of Liu et al., (1994) in which 300 µL protoplasts in MC solution were mixed with 10-20 µg plasmid DNA (each pK7RWG2/ALs vector), 300 µL 40% PEG solution and 4 mL PSM solution (MS medium with 0.44% (w/v) vitamins, 2% (w/v) sucrose, pH 5.7). Finally, protoplasts were incubated for 18-24 h in darkness at room temperature before being visualized under an inverted epifluorescence microscope Olympus IX70 using the FITC filter for GFP (450-490 nm) and Cy3 filter for RFP (530-560 nm).

Actinidia deliciosa somatic organogenesis

The explants (that come from the transformation process) were deposited in Petri dishes with medium I (MS salts 2.2 g/L, vitamins 0.22%, sucrose 3%, myoinositol 0.01%, pH 5.8, supplemented with 2,4-D 1mg/L, BAP 0.5 mg/L, Kanamycin 20mg/L and Timentin 300mg/L). The explant were subculture every 3 weeks in the same media for 6 to 8 weeks under photoperiod conditions of 16 hours of light and 8 hours of darkness at 24°C until calli induction. Subsequently, the explants were transferred to Petri dishes with medium II (MS salts 2.2 g/L, vitamins 0.22%, sucrose 3%, myoinositol 0.01%, pH 5.8, supplemented with BAP 5 mg/L, Kanamycin 40 to 100mg/L and Timentin 200mg/L, where they were kept for 4 to 6 months under a photoperiod of 16 light hours and in constant replacement of the culture medium, until the appearance of shoots. Then, the calli with buds were transferred to Petri dishes with medium III (MS salts 2.2 g/L, vitamins 0.22%, sucrose 3%, myoinositol 0.01%, pH 5.8, supplemented with BAP 0.5 mg/L, Kanamycin 100mg/L and Timentin 100mg/L) for 4 to 6 weeks, favoring the elongation of the buds, thus facilitating their individualization and multiplication. In case of ex vitro transplanting, the shoots were left in medium IV (MS salts 2.2 g/L, vitamins 0.22%, sucrose 1.5%, myoinositol 0.01%, pH 5.8, supplemented with IBA 0.5mg/L, Kanamycin 100mg/L and Timentin 100mg/L) until the appearance of roots and then acclimatized in the greenhouse.
